# Molecular identification and phylogenetic analysis of chikungunya virus among dengue-negative patients in Kolkata, India

**DOI:** 10.1371/journal.pone.0301644

**Published:** 2024-04-04

**Authors:** Rajendra Prasad Chatterjee, Aroni Chatterjee, Sabbir Ansari, Shilpa Chatterjee, Shyamalendu Chatterjee, Nilanjan Chakraborty

**Affiliations:** 1 ICMR-National Institute of Cholera & Enteric Diseases, Kolkata, India; 2 Department of Biomedical Science, Chosun University College of Medicine, Gwangju, Republic of Korea; University of South Florida, UNITED STATES

## Abstract

Dengue and chikungunya are co-circulating vector-borne diseases that share a significant number of clinical symptoms. To identify variables to aid physicians in making rapid and effective diagnostic decisions, we performed molecular diagnosis of the chikungunya virus and examined the clinical manifestations of chikungunya cases to identify the prevalence among dengue-negative individuals in Kolkata. Dengue suspected patients’ samples were collected during January 2020—December 2021 and Enzyme-linked immunosorbent assay (ELISA) and reverse transcription-polymerase chain reaction (RT-PCR) methods have been performed to confirm the prevalence of chikungunya infection among dengue-negative patients. By performing phylogenetic analysis, comparing clinical classifications, identifying disease aetiology using clinical and laboratory factors, and evaluating the time course of several clinical variables, we have evaluated the clinical manifestations linked to dengue and chikungunya virus infections. Chikungunya infection was found in 15.1% and 6.3% of the 635 dengue-negative patients, as determined by ELISA and RT-PCR, respectively. Arthritis and myalgia were more common in chikungunya-infected patients at the time of hospital admission while conjunctivitis, photosensitivity, arthralgia, Anorexia, fatigue, retro-orbital pain, vomiting, dermatitis, or swollen glands were significantly presented as an overlapping symptom. Although dengue and chikungunya infections have significant clinical overlap, basic clinical and laboratory criteria can predict these diseases at presentation for proper management. Effective management enables doctors to treat and care for patients properly and contributes to the development of control measures for these infections in a medical setting.

## 1. Introduction

Arthropod-borne chikungunya virus (CHIKV), which is responsible for Chikungunya fever, is a positive sense ribonucleic acid (RNA) virus with a genomic size of 11.8 kb and is a member of the *Togaviridae* family [1]. CHIKV infection is a mosquito-borne viral disease that cause fever, acute febrile illness, rash, and arthralgia [2]. Following its comeback since 2006, Chikungunya fever has turned into a global health issue [3].

In 2006, Kolkata, West Bengal, India, experienced a CHIKV out-break. Even though a few occasional cases of CHIKV infection were investigated from a few Indian states between 2010 and 2015 [3], the rate of infection has been declining until a 2016 outbreak in the capital city Delhi [4]. Currently, the disease has spread throughout the entire nation and outbreaks cause significant economic and productivity losses. Due to the shared vector and clinically comparable symptoms between dengue and chikungunya virus, coinfections have also been reported [4]. Despite having identical symptoms, these two diseases have quite distinct outcomes and treatment plans. To begin the proper treatment and prevent complications including haemorrhages, acute respiratory distress syndrome (ARDS), renal failure, and arthritis, clinicians must develop a differential diagnosis based on a variety of clinical presentations and lab testing [5]. Moreover, the CHIKV has caused a significant amount of morbidity in India in recent years, but the true disease burden may be underestimated because of insufficient precise reporting, which would augment it significantly. Since there is no other way to treat or prevent CHIKV infection, it is crucial to get a precise diagnosis to assess the disease burden and implement therapeutic and control measures. The favoured approaches for diagnosing recent CHIKV infection include the detection of viral genomic RNA and IgM antibodies; gene amplification assays are only useful in the early stages, that is, only within 2–6 days after the onset of disease when the virus is still in circulation [6]. Further, the development of MAC ELISA assay has provided a rapid and reliable technique for the IgM detection [7].

In our lab, a sizable portion of dengue virus (DENV) negative symptomatic samples go undetected each year. Due to the dearth of literature on prevalence, clinical profile, and aberrant manifestations of the chikungunya from Eastern India, we performed molecular diagnosis and phylogenetic analysis of CHIKV and analysed various clinical manifestations of chikungunya cases to determine the prevalence of CHIKV infection among symptomatic DENV negative individuals, if any.

## 2. Materials and methods

### 2.1. Study area

This retrospective study was conducted at Virus Laboratory, Indian Council of Medical Research (ICMR)-National Institute of Cholera and Enteric Diseases, Kolkata, India, during January 2020—December 2021. This study was approved by the Institutional Ethical Committee vide Institutional memo no: ICMR/VU/57-DBT Chikungunya Project, dated: 05/01/2017. Well informed permission was acquired from discrete contributor included in this study. Written consent to publish has been received from the participant.

### 2.2. Sample collection

Samples were collected from the dengue suspected cases and among them, total 635 DENV negative cases were included in this study for further investigation of CHIKV infection. The inclusion criteria for these specimens were followed the World Health Organization (WHO) guidelines [8]. Along with fever, any three of these symptoms were believed to be indicators of CHIKV infection, such as headache, enteralgia, conjunctivitis, photo sensitivity, nausea, vomiting, anorexia, dermatitis, arthralgia, and myalgia, with or without any haemorrhagic manifestation. The study was planned to be non-discriminatory in terms of age or gender. Patients who visited the care units with illnesses other than febrile illnesses and patients who declined to participate in the study were both excluded from it. Approximately, 2-3ml of blood samples was collected by the laboratory personnel from the patients having fever along with other possible history of illness from the fever clinic unit of different medical colleges and hospitals. Along with the blood samples, clinical and demographical data of patients were collected by the health care workers at the time of patients’ visit and sent to our laboratory for further analysis. Maintaining cold chain, all the samples were transported to our virology laboratory for the confirmation of the infection, if any. The centrifugation was used to isolate serum from the specimens at 3000g for 10 min at 4°C. Serum were stored for serological and molecular tests at -80° C in aliquots, until further used.

### 2.3. DENV diagnosis

DENV infection was identified by using the non-structural protein 1 (NS1) Antigen Rapid Test (SD Bioline, Seoul, Korea), and PanBio Dengue immunoglobulin M (IgM) Capture Enzyme linked immunosorbent assay (ELISA) (PanBio Kit, Alere, Waltham, MA, USA). The sensitivity and specificity of the SD Bioline DENV antigen test was 92.4% and 98.4% respectively and for the PanBio DENV Capture ELISA kit, it was 94.7% and 100% respectively. A total of 635 cases were found to be clinically diagnosed as dengue but were negative for the presence of DENV either by NS1 antigen or IgM ELISA. Therefore, to further determine the aetiology of the DENV-negative samples, we performed serological methods and RT-PCR method for the diagnosis of CHIKV infection, if any.

### 2.4. Serological detection of CHIKV

The serological detection of CHIKV infection was carried out to detect the chikungunya specific IgM antibody in serum using in-house chikungunya IgM antibody capture ELISA kit (MAC ELISA, National Institute of Virology, Pune, India) and SD BIOLINE Chikungunya IgM test kit (Standard Diagnostics Inc, Gyeonggi-do, South Korea) based on the availability of the kit. The diagnostic sensitivity and specificity of the CHIKV IgM MAC ELISA kit was 95% and 98% respectively and for the SD BIOLINE Chikungunya IgM test kit, it was 97.1% and 98.9% respectively. All the assay was performed and optical density (OD) was measured at 450nm using the kit specific protocol provided by the manufacturer with the help of semi-automatic ELISA micro-plate reader and washer made by Thermo Fisher Scientific (India).

### 2.5. Molecular detection of CHIKV

After excluding 96 CHIKV IgM ELISA positive samples, the remaining 539 IgM ELISA negative samples were used for RNA isolation using a manual QIAamp viral RNA isolation kit (made by Qiagen, GmbH, Hilden, Germany) according to manufactures’ instruction. After RNA extraction, a one-step RT-PCR targeting the CHIKV envelop 2 (E2) gene was performed using commercially available RT-PCR kit from Chromous Biotech (Chromous Biotech Pvt. Ltd, Bangalore, India). The cross reactivity of the primers has been thoroughly checked using in-silico analysis and molecular biology method. The primer sequences used in the template formation were CHIKV-FP (5’-TGTAAGAACATCAGCACCGTGTACG-3’) and CHIKV-RP (5’CAAGTTCAGCATTACGGGACCA-3’). The assay was carried out in a Bio-Rad thermal cycler, starting with the cDNA synthesis (42°C for 15 min), followed by initial denaturation (94°C for 30 sec), and finally 35 PCR cycles, which include denaturation at 94°C for 30 sec, annealing at 50°C for 30 sec, and expansion at 72°C for 90 sec. The final elongation stage was fixed out for 7 minutes at 72°C. The final PCR products were run in a gel electrophoresis system (made by Bio-Rad) for 45–60 minutes at 80–100 volts in a 2% agarose gel before being exposed to UV light in a gel imager. For each positive sample, the amplified products were seen as bands that were approximately 490 base pair (bp) in size.

### 2.6. Nucleotide sequencing and phylogenetic analysis

14 samples that produced a prominent band out of the 34 RT-PCR positive samples were sequenced using a large dye terminator sequencing kit and an ABI 3500 XL genetic analyser. The partial nucleotide sequence of the E2 gene obtained from the isolates of the present study were compared with other CHIKV strains reported from different geographic regions including India and Africa (**S1 Table**). Using Basic Local Alignment Search Tool (BLAST) techniques, nucleotide homology percentages were calculated and the most comparable sequences of the CHIKV E2 gene were found [9]. To identify the evolutionary relationship, phylogenetic analysis was performed. The neighbor-joining method with Kimura-2-parameter model served as the foundation for the phylogenetic tree [10]. 1000 bootstrap replications of the MEGA X software programme were used to estimate the initial Maximum Likelihood (M-L) strategy using pair wise distance [11].

### 2.7. Isolation of virus

For many years, virus isolation was the gold standard for CHIKV diagnosis; however, it is no longer frequently employed in routine diagnosis [12]. Between 2 and 6 days after the start of the disease, high titres of the virus are discernible in serum or plasma samples from the patient. But starting on day 5 after the disease begins, the viral load begins to decline. As a result, only the acute stage of the infection is when the test is effective for diagnosing purposes. In this investigation, among 34 RT-PCR positive samples, 20 samples those exhibited weak band on the agarose gel electrophoresis were further chosen for virus isolation to confirm its true positivity for CHIKV infection. A positive control strain (EF027140.1) was obtained from ICMR-National Institute of Virology for this study. Confluent monolayer of Vero cells was infected with CHIKV at a multiplicity of infection (MOI) of 0.1. Growth medium removed from the Vero cells in 24 well plates and 0.1 ml of the sample, diluted 100-fold, inoculated into the wells. The suspension was allowed to adsorb for 2 hours at 28°C in an incubator with 5% CO2.The wells were washed with 1% PBS and then Eagle’s minimal essential medium (MEM) was supplemented with 10% FBS and antibiotic mixture. Afterwards wells were incubated under the same condition overnight in an incubator. For a week, all the cell culture wells and flasks were examined under in-verted microscope (Zeiss 40x) daily for the arrival of cytopathic effect (CPE), if any. Upon presence of the CPE, cell culture solution was subjected to separate cell detritus by spinning the solution using centrifugal force at 1000g for 12 mins at 4°C. The supernatant was aliquoted and stored for the RNA isolation.

### 2.8. Statistical analysis

For descriptive analyses, cross tabulation was used to express categorical variables. Discrete variables were first tested for normalcy, normalized as needed, and then averages were analysed by one-way Analysis of variance (ANOVA). A 2 x 2 contingency chi square analysis was performed to find association between CHIKV infections with different clinical symptoms. Signs/symptoms and underlying medical conditions for the group of patients were compared using chi-square or Fisher’s exact tests. Odds ratios and 95% confidence interval (CI) values were calculated for each of the common symptoms. Statistical analyses were performed using IBM SPSS Statistics for Windows, version 22.0 (IBM Corp., Armonk, NY).

## 3. Results

### 3.1. Demographic distribution

**Fig 1:**
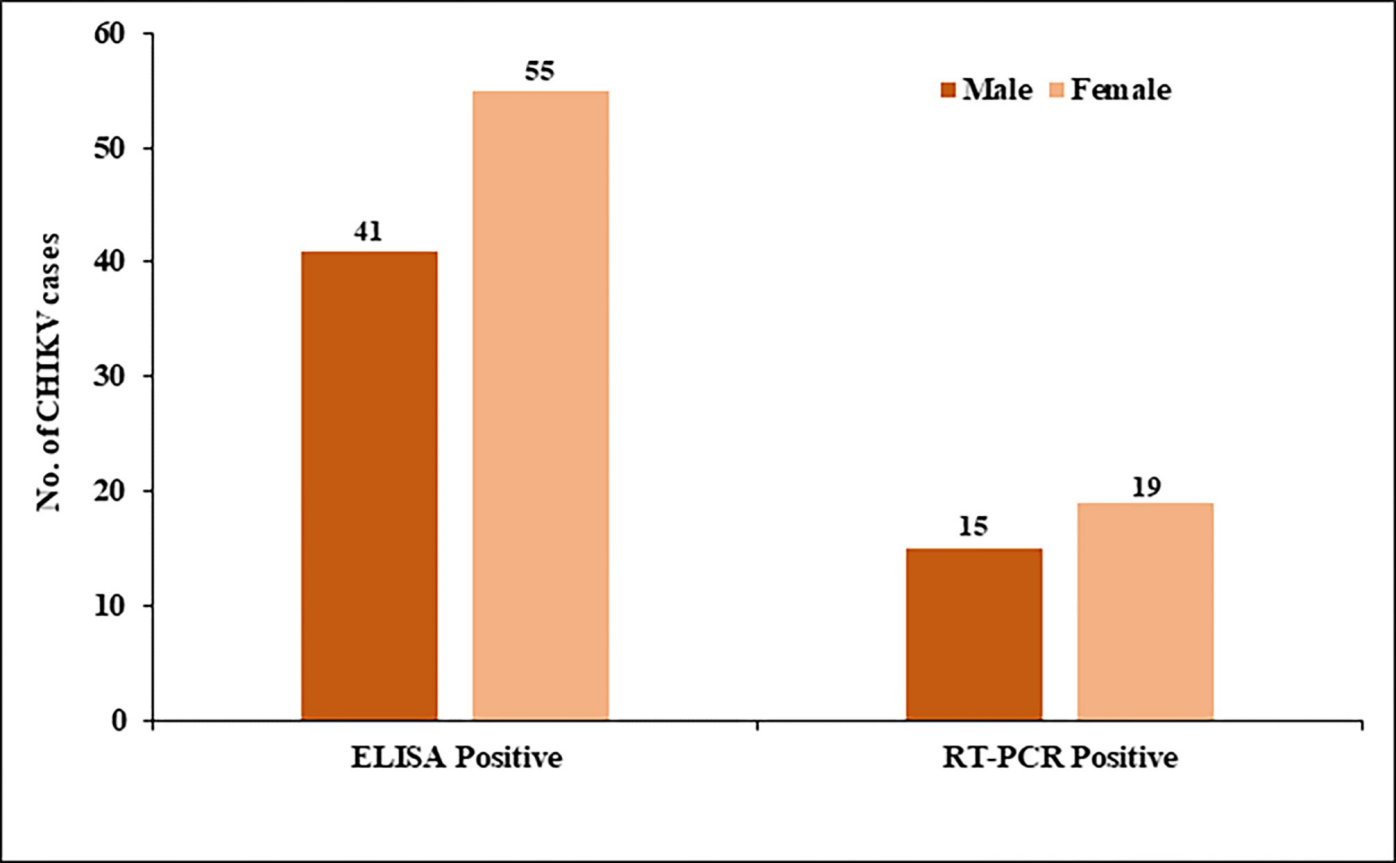
Gender-wise distribution of CHIKV cases based on ELISA and RT-PCR.

We investigated samples of total 635 Dengue-negative patients from all age categories, including 301 men and 334 females. According to our research into the CHIKV incidence, 15.1% (96/635) were found to have the CHIKV infection confirmed by ELISA and 6.3% (34/539) confirmed by RT-PCR. Male patients’ CHIKV seropositivity was lower than that of female patients’ which is 42.7% (41/96) in ELISA and 44.1% (15/34) in RT-PCR. Female patients had a higher percentage of CHIKV seropositivity such as 57.3% (55/96) and 55.8% (19/34) than male patients based on ELISA and RT-PCR respectively. Most CHIKV-related infections in both sexes occurred in patients between the ages of 31 to 40 [**Fig 1**].

**Fig 2:**
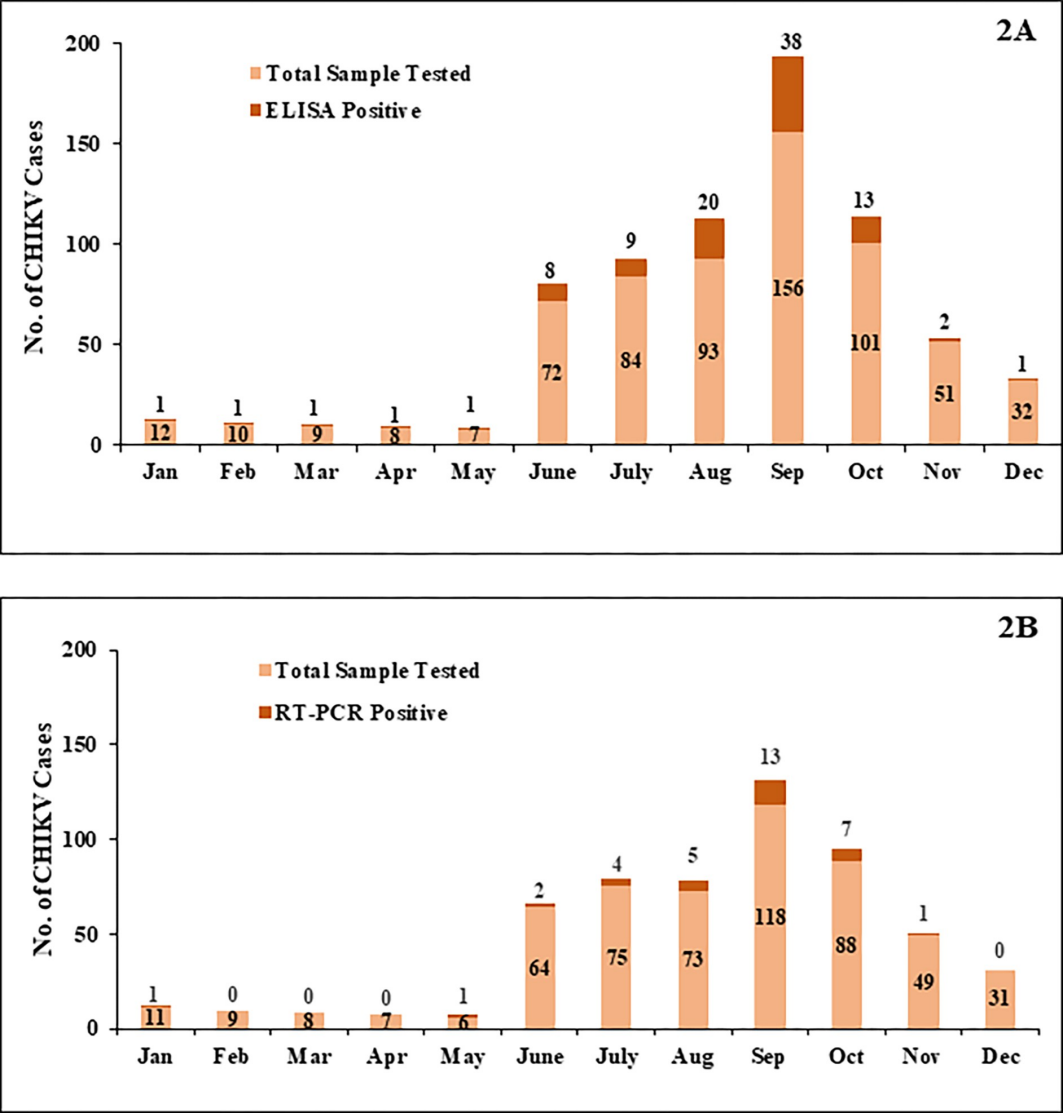
Month-wise distribution of CHIKV cases based on ELISA (2A) and RT-PCR (2B).

Further, we analysed the month-wise distribution of CHIKV cases based on ELISA (2A) and RT-PCR (2B). The study found that CHIKV infection rates in September were highest among other months showing positivity of 24.4% (38/156) in ELISA and 11% (13/118) in RT-PCR. [**Fig 2A and 2B**].

In this figure, we analysed the month wise prevalence rate of CHIKV by considering the total number of CHIKV sample tested and the samples showed positive results in both ELISA and RT-PCR assay. The samples showed ELISA positive results for CHIKV were not considered further for RT-PCR assay.

**Fig 3:**
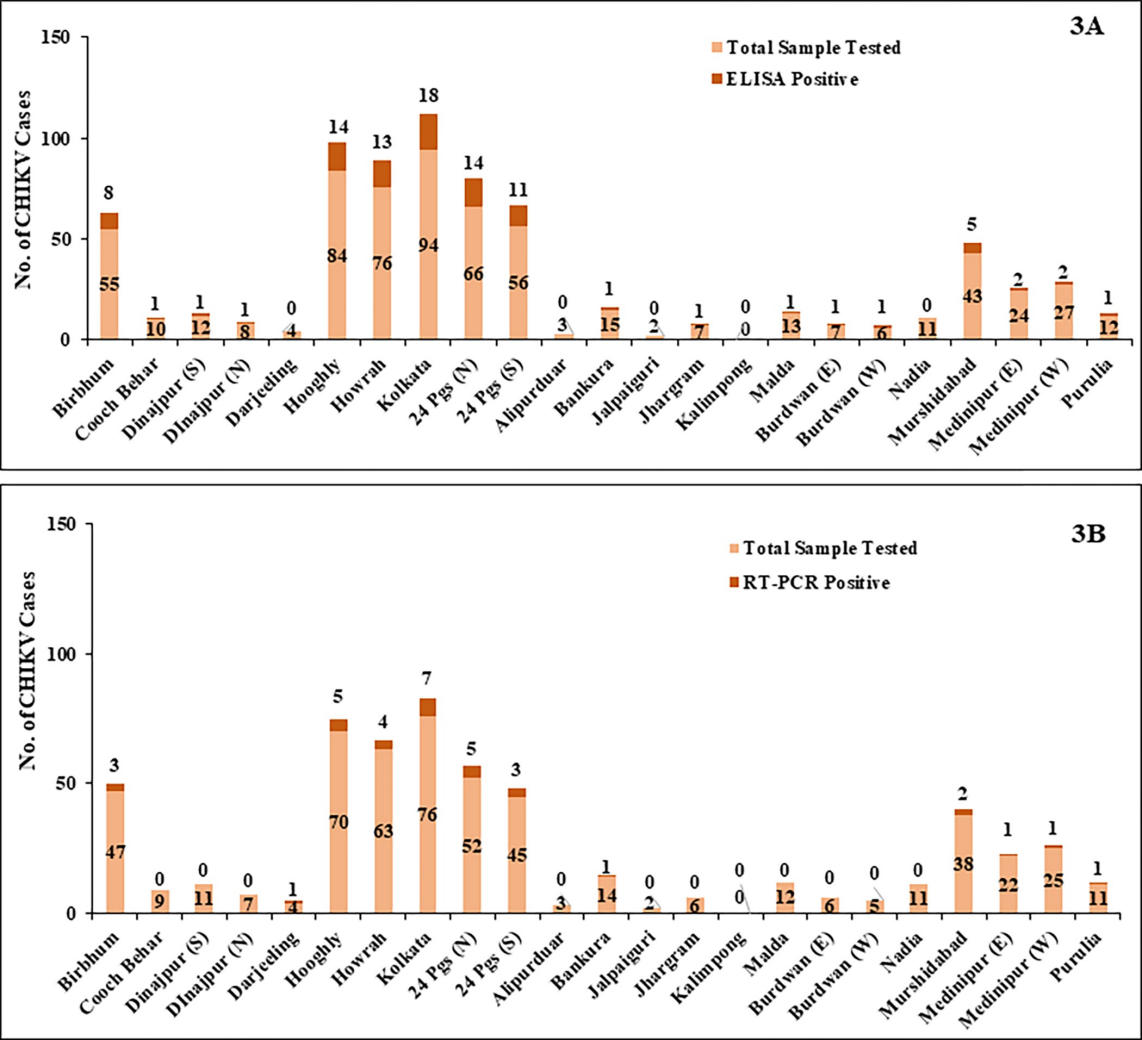
District-wise distribution of CHIKV cases based on ELISA (3A) and RT-PCR(3B). In this figure, we have analysed the district-wise prevalence rate of CHIKV by considering the total number of CHIKV sample tested and the samples showed positive results in both ELISA and RT-PCR assay. The samples showed ELISA positive results for CHIKV were not considered further for RT-PCR assay.

In addition to that, the distribution pattern of the CHIKV infection in Kolkata and surrounding districts were analysed to identify the diverse nature of the disease. The effects of the CHIKV infection were obviously noted in Kolkata and its surrounding districts, including Hooghly, S24 Parganas, Howrah and N24 Parganas in both ELISA and in RT-PCR [**Fig 3A and 3B**].

### 3.2. Phylogenetic analysis

**Fig 4:**
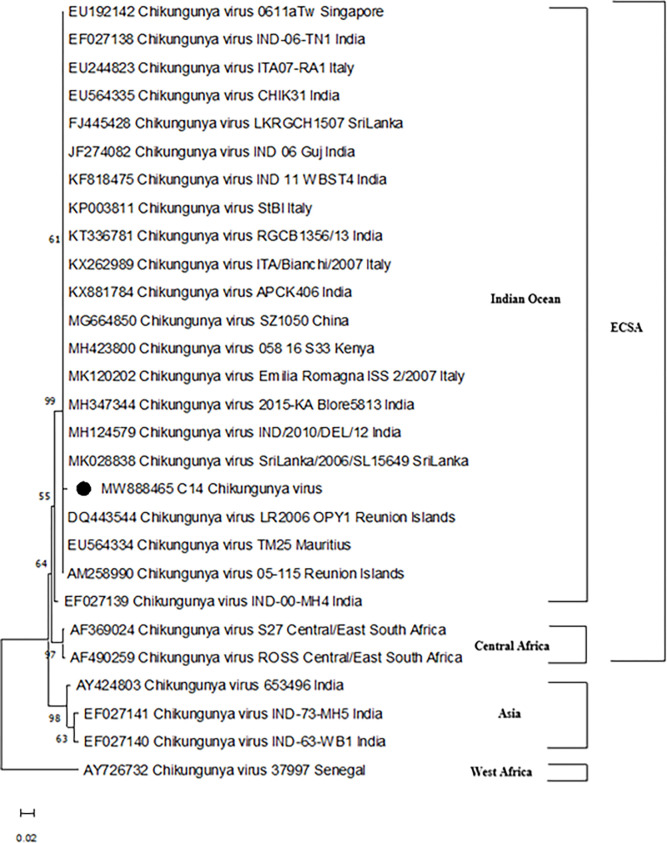
Phylogenetic tree was constructed based on the envelope gene (~ 490 bp) of CHIKV sample (●) and available virus sequences from GenBank. The tree was inferred by using the Maximum Likelihood method and Kimura-2-parameter model. The tree is drawn to scale, with branch lengths measured in the number of substitutions per site. To evaluate replicated tree confidence, 1000 bootstrap replicates were performed. Evolutionary analyses were conducted in MEGA X. This analysis involved 28 nucleotide sequences.

Phylogenetic analysis based on the nucleotide sequence of the partial E2 gene (~490 nucleotides) of CHIKV-positive isolate was analysed and compared with other globally diverse CHIKV isolates. The genetic diversity and evolutionary linkages of CHIKV envelope gene sequences and virus sequences from GenBank were evaluated and shown in **S1 Table**. The phylogenetic tree analysis that resulted revealed that the identified Kolkata strain (GenBank Accession Number: MW888465) was clustered with the East Central South African (ECSA) genotype (Indian Ocean lineage) and did not cluster with the Asian strains that caused 1973, and 2003 CHIKV outbreak in India [**Fig 4**]. In addition, the identified Kolkata strain in our study showed more genetically similar relationship with the 2006 Chikungunya virus Sri Lanka strain [**Fig 4**]. Moreover, the phylogenetic tree revealed that 2010 and 2015 India strains isolated from Delhi and Bangalore respectively, are genetically close to our identified study isolate. Further, the identified Kolkata strain was clustered with many Indian origin CHIKV strains [**Fig 4**].

### 3.3. Cell culture assay

While performing the cell culture assay, approximately 7–9 days post infusion, the Vero cell line showed cytopathic effect (CPE) in response to 20 RT-PCR positive samples which showed weak band on the agarose gel electrophoresis. An uninfected patient sample and an infected patient sample are shown in the Vero cell line’s microscopic image [**S1 Fig**].

### 3.4. Statistical analysis

Furthermore, we performed the statistical analysis using ANOVA based on mean weight, blood sugar level and age of patients tested for CHIKV infection using both RT-PCR and ELISA. Following are the characteristics of patients who were tested for CHIKV infection using RT-PCR method. The mean age of the patients was 46 ± 13.9 and mean body weight (kg) was 64 ± 9.7 showing average blood sugar (mg/dL) level of 125 ± 23.9 but had no statistically significant differences between the CHIKV negative and positive RT-PCR patients based on the ANOVA results [**Table 1**].

**Table 1:** Mean weight, blood sugar level and age of patients based on the RT-PCR and serological detection of CHIKV.

Factors	RT-PCR	P Value	ELISA	*P* value
Age	46 ± 13.9	0.0	42 ± 13.3	0.5
Weight (Kg)	64 ± 9.7	0.2	63 ± 9.7	0.8
Blood Sugar (mg/dL)	125 ± 23.9	0.5	97 ± 22.3	0.0

Moreover, we observed no statistically significant difference between the CHIKV IgM ELISA positive and negative patients in the mean weight (kg) count, age, and blood sugar level based on the ANOVA results [**Table 1**]. Days of illness or onset of illness are crucial indicators that the acute CHIKV infection can be found using the RT-PCR method. The first day of the illness up to the fifth day after infection is the best time-frame to look for the specific CHIKV gene using RT-PCR. IgM ELISA, on the other hand, showed the opposite pattern in the identification of CHIKV infection. On and after the sixth day following the onset of the dis-ease, it was able to discover anti-chikungunya IgM antibodies. Most symptoms subside after a week, however arthralgia persisted for several months in the majority of CHIKV positive individuals. No deaths have been reported among the CHIKV-positive patients we have followed up with. Further detailed analysis of the clinical symptoms showed that headache, nausea, myalgia, arthralgia, dermatitis, and fever were more prominent symptoms associated with CHIKV positive patients identified by both ELISA and RT-PCR assay [**Tables 2** & **3**].

**Table 2:** Determination of frequency and odds ratios (OR) analysed based on different clinical symptoms for chikungunya IgM positive samples.

Clinical symptoms	IgM Positive No. (%)	IgM Negative No. (%)	OR (95% CI)
Arthralgia	78 (81.2%)	43 (7.8%)	49.9
			(27.4 to 91.07)
Dermatitis	58 (60.4%)	178 (33.0%)	3.09
			(1.98 to 4.84)
Myalgia	61 (63.5%)	217 (40.2%)	2.59
			(1.65 to 4.06)
Vertigo	11 (11.4%)	31 (5.75%)	2.12
			(1.03 to 4.38)
Anorexia	14 (14.6%)	45 (8.3%)	1.87
			(0.98 to 3.57)
Retro-orbital Pain	4 (4.1%)	15 (2.8%)	1.52
			(0.49 to 4.68)
Fever	91 (94.8%)	498 (92.4%)	1.49
			(0.58 to 3.89)
Nausea	19 (19.8%)	93 (17.2%)	1.18
			(0.68 to 2.05)
Cough & Cold	1 (1.0%)	5 (0.9%)	1.12
			(0.13 to 9.73)
Sore Throat	7 (7.3%)	45 (8.3%)	0.86
			(0.38 to 1.98)
Enteralgia	17 (17.7%)	111 (20.6%)	0.83
			(0.47 to 1.46)
Vomiting	3 (3.1%)	28 (5.2%)	0.59
			(0.17 to 1.98)
Headache	12 (12.5%)	122 (22.6%)	0.48
			(0.26 to 0.92)
Seizure	0 (0.0%)	0 (0.0%)	0 (—)

**Table 3:** Determination of frequency and odds ratios analysed with different clinical symptoms for chikungunya RT-PCR positive results among IgM negative samples.

Clinical symptoms	RTPCR Positive No. (%)	RTPCR Negative No. (%)	OR (95% CI)
Arthralgia	31 (91.2%)	12 (2.3%)	62.14
			(18.5 to 208.7)
Dermatitis	29 (85.2%)	149 (29.5%)	13.8
			(5.26 to 36.5)
Myalgia	28 (82.3%)	189 (37.4%)	7.8
			(3.17 to 19.19)
Enteralgia	19 (55.9%)	92 (18.2%)	5.68
			(2.78 to 11.6)
Cough & Cold	0 (0.0%)	5 (1%)	2.9
			(0.39 to 21.9)
Nausea	10 (29.4%)	83 (16.4%)	2.19
			(0.97 to 4.6)
Vertigo	3 (8.8%)	28 (5.5%)	1.65
			(0.47 to 5.7)
Anorexia	4 (11.8%)	41 (8.1%)	1.5
			(0.5 to 4.49)
Retro-orbital Pain	1 (3.0%)	14 (2.8%)	1.06
			(0.13 to 8.34)
Sore Throat	2 (5.9%)	43 (8.5%)	0.67
			(0.15 to 2.89)
Vomiting	1 (3.0%)	27 (5.3%)	0.54
			(0.07 to 4.07)
Headache	4 (11.8%)	118 (23.3%)	0.44
			(0.15 to 1.27)
Fever	33 (97.0%)	464 (91.9%)	0.44
			(0.15 to 1.27)
Seizure	0 (0.0%)	0 (0.0%)	0 (—)

### 3.5. Clinical features and overlapping symptoms

**Fig 5:**
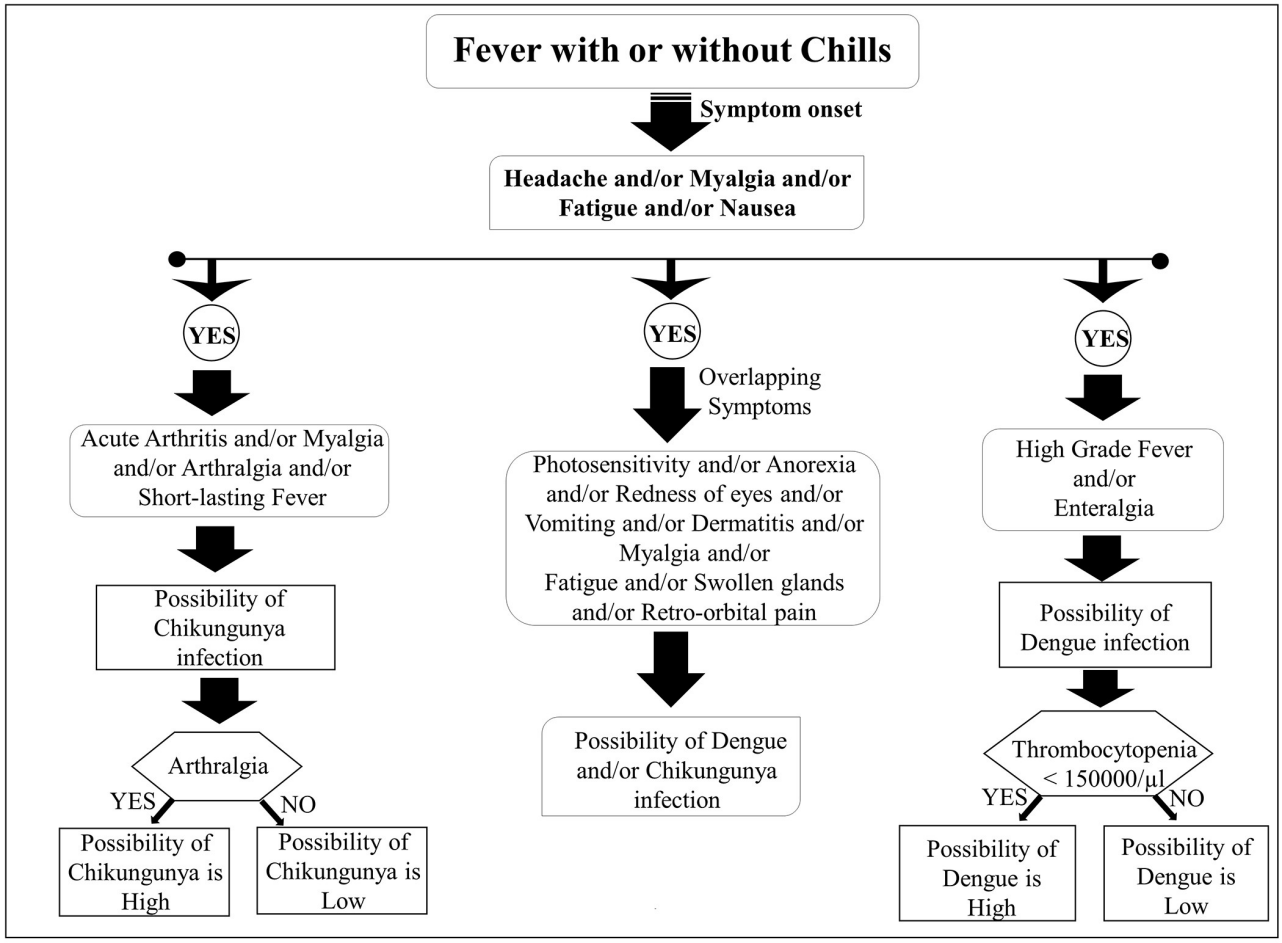
Schematic diagram showing the significant clinical features and major overlapping symptoms among chikungunya and dengue infected patients.

Additionally, significant clinical features and major overlapping symptoms among chikungunya and dengue infected patients were analysed. Symptoms such as high-grade fever, enteralgia, and thrombocytopenia were more common in dengue patients, while Chikungunya fever patients were more likely to experience acute arthritis, short-lasting fever, joint swelling, and myalgia/arthralgia [**Fig 5**].

## 4. Discussion

Chikungunya epidemics have been documented on the Indian subcontinent since the early 20th century [2, 13]. Not only in Indian sub-continent, but also in other continent such as, East-Central South African, American region, cases of simultaneous infections involving different arboviruses are becoming common which increases the chance of infection in those areas [14]. In this study, the seasonal distribution of CHIKV was analyzed which indicated a high prevalence during the monsoon season with positive cases being raised gradually from June with peak in the month of September. This finding correlated with several other findings reported in India [15–21]. This may be due to increased transmission of disease at the start of the rainy season with increased breeding of both *Aedes aegypti* and *Aedes albopictus*. The seasonal peaks highlight the importance of raising public awareness and implementing essential measures to control vectors, such as enhancing sanitation and hygiene before the monsoon season. Furthermore, we anticipate that this data will enhance clinical diagnosis, offering a valuable perspective for enhancing public health management.

Every year, samples with high to moderate fever, photosensitivity, nausea, anorexia, conjunctivitis, and in most of the cases weakness for at least one week but no evidence of the DENV were chosen for this investigation. Our study confirms that RT-PCR is the most reliable approach for confirming active CHIKV infection in individuals with 1–5 days of clinical illness by acting to detect the CHIKV specific genes. We discovered a large overlap between the signs and symptoms of chikungunya patients and dengue positive individuals. They have little ability to distinguish between diagnosis because many of these traits considerably overlap and there is substantial heterogeneity among individuals who have the same ailment. Like a prior study, the changes in platelet counts that are most apparent at presentation [22]. Myalgia/arthralgia, which were more commonly present in chikungunya patients whereas thrombocytopenia was more frequently seen in dengue positive cases. Cases among patients from the Peruvian coast reported by Luis et al. also mentioned that myalgia/arthralgia were most common among CHIKV infected patients [23].

Moreover, co-circulation of zika virus, DENV, and CHIKV poses significant challenges in accurately diagnosing these infections due to overlapping signs and symptoms, especially in regions like India where zika virus is present. Considering the potential cross-reactivity in diagnostic assays targeting specific viruses is crucial. Although India’s environment favours zika virus transmission due to *Ae*. *aegypti* mosquitoes, reported zika cases are scarce, yet the risk of misdiagnosis with other arboviruses necessitates precise diagnostic tools [24, 25]. Notably, there is currently no reported evidence of zika virus infection among patients from West Bengal, the region from which our samples were collected.

To assist doctors in differentiating between these two infections for effective triaging for site of care and clinical management, diagnostic and prognostic algorithms with significant negative predictive values are advised. It is crucial to recognise dengue patients because they require intensive monitoring and treatment planning in a hospital, as they may produce severe haemorrhagic fever, which may not be treated as an outpatient. According to our study, basic clinical and laboratory criteria can predict these infections at presentation and categorise them into symptoms and laboratory tests for appropriate care. The platelet level cut-offs between dengue and chikungunya infected patients also show a substantial variation. Since there are no specific antivirals or treatment options for chikungunya, the main stay of patient management is symptomatic treatment. To prevent dehydration, the patient is typically encouraged to get enough rest and drink a lot of water. Until dengue infection has been ruled out, non-steroidal anti-inflammatory medicines should not be taken. To treat pain and fever, usually acetaminophen or paracetamol is used.

CHIKV is a dynamic disease with the potential to possess single point mutation [3]. Continuous surveillance of the phylogeny and mutations of the circulating viral strain of CHIKV is crucial to prevent and manage future epidemics. Here, we reported a simultaneous circulation of different CHIKV Kolkata strain (MW888465) among patients which showed ancestral similarity with the previously reported Sri Lankan CHIKV strain belonging to the ECSA genotype. According to the previous studies, as India has witnessed a shift of CHIKV genotype from Asian in 1963 and 1973 to ECSA in 2005–2006, vigilance is required to keep watching for any possible shift in future which may have epidemic potential [26].

Recent studies reported that CHIKV exist as a co-infection within 10% of DENV positive patients [4]. Brooks et al. also reported the occurrence of acute illness along with neurological findings in a Brazilian patient who was coinfected with CHIKV and DENV [27]. Further, the potential cross-reactivity of IgM antibodies with other alpha viruses is an important consideration in the context of diagnostic assays. Despite having limitations, using IgM antibodies alone for determining recent infection with chikungunya virus (CHIKV) is common practice due to its rapid turn-around time, easy epidemiological surveillance, extended diagnostic window period, widespread use and familiarity and accessibility in resource-limited setting. Here, IgM capture ELISA detection kit used that has already been proven to be specific monoclonal antibody for the specific chikungunya antigens by multiple studies worldwide, having 99.3% serological sensitivity and 99.9% serological specificity. Furthermore, IgM kits from multiple sources used for sensitive validation of our finding in our laboratory for cross reactivity.

There are currently no licenced vaccinations or antivirals available for both the diseases, but India has started several initiatives including using traditional remedies [3, 28]. For the proper diagnosis with the same type of disease expressions, recommendations are made for better treatment and attention should be given to follow up with the patients. Thus, it will help us to get the actual number of CHIKV infections in a particular area and to examine variations in symptoms, if any. Our study revealed that many dengue negative patients overlooked and left untreated, but they may have suffered due to CHIKV infection, which is why it is important to identify the actual disease so that patients can get rid of from the mortality and morbidity of CHIKV infection. Overlapping of symptoms may be acquired due to mutational adaptation and further in-depth study required on that which will help community to get the actual number of CHIKV infections in a particular area and to examine variations in symptoms, if any.

## 5. Conclusion

This study suggests that, doctors should offer CHIKV testing for febrile dengue-negative patients who are displaying similar clinical symptoms Our results suggest one of the possibilities for why CHIKV infections are less common in West Bengal and other Indian states. To determine and the actual length of the CHIKV infection and to diagnose patients swiftly, offer the appropriate care, and follow-up, the state health department must carry out meticulous monitoring. Our research has demonstrated that there is significant clinical presentation overlap between these illnesses. The ability of doctors to anticipate these infections using straightforward clinical and laboratory characteristics has also been demonstrated by our research, allowing for more effective management of CHIKV infection in a region endemic to DENV infections.

## Supporting information

S1 Table Background information of selected GenBank reference strains used in this study. (DOCX)

S1 Fig Image of an uninfected patient sample and an infected patient sample are shown in the Vero cell line’s microscopic image. (TIF)
