# Supplementary material for: Molecular identification and phylogenetic analysis of chikungunya virus among dengue-negative patients in Kolkata, India
Source: PLoS One. 2024 Apr 4;19(4):e0301644. doi: 10.1371/journal.pone.0301644 (PMC10994276; doi:10.1371/journal.pone.0301644)
Supplement: S1 Table — (DOCX) [file pone.0301644.s001.docx]

| **Isolates** | **Origin** | **Year** | **Source** | **Accession Numbers** |
| --- | --- | --- | --- | --- |
| IND/2010/DEL/12 | India | 2019 | Human | MH124579 |
| SriLanka2006/SL15649 | Sri Lanka | 2019 | Human | MK028838 |
| 2015-KA_Blore5813 | India | 2018 | Human | MH347344 |
| Italy/Emilia Romagna-ISS-2/2007 | Italy | 2018 | Human | MK120202.1 |
| 058_16_ S33 | Kenya: Mandera | 2018 | Human | MH423800 |
| SZ1050 | China | 2017 | Human | MG664850 |
| APCK406 | India | 2016 | Human | KX881784 |
| USA/91064A/2006 | USA | 2017 | Human | KY575568.1 |
| ITA/Bianchi/2007 | Italy | 2016 | Human | KX262989.1 |
| RGCB1356/13 | India: Kerala | 2015 | Human | KT336781 |
| StBI | Italy | 2014 | Human | KP003811 |
| IND-11-WBST4 | India | 2017 | Human | KF818475.1 |
| IND-06-Guj | India | 2012 | Human | JF274082.1 |
| LKRGCH1507 | Sri Lanka | 2009 | Human | FJ445428 |
