# Supplementary figures and images for: Molecular identification and phylogenetic analysis of chikungunya virus among dengue-negative patients in Kolkata, India
Source: PLoS One. 2024 Apr 4;19(4):e0301644. doi: 10.1371/journal.pone.0301644 (PMC10994276; doi:10.1371/journal.pone.0301644)

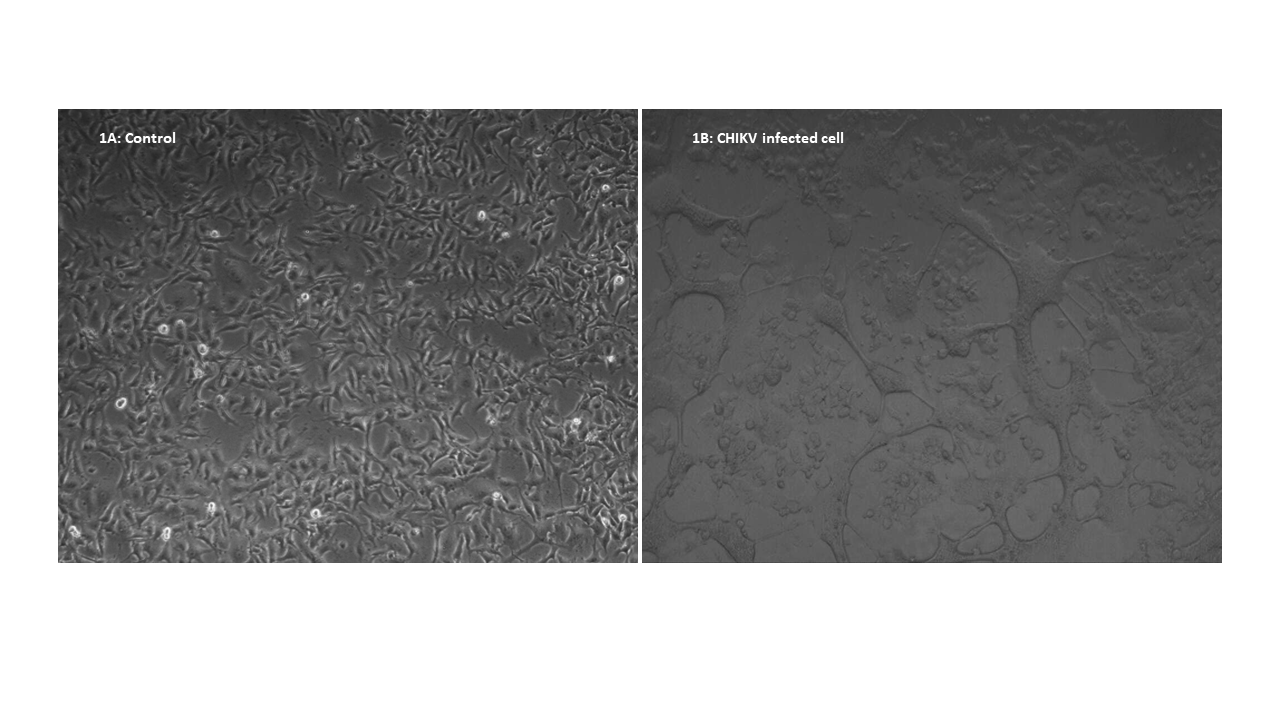

Supplement: S1 Fig — (TIF) [file pone.0301644.s002.tif]
